# Supplementary material for: Lethality of SARS-CoV-2 infection in K18 human angiotensin-converting enzyme 2 transgenic mice
Source: Nat Commun. 2020 Nov 30;11:6122. doi: 10.1038/s41467-020-19891-7 (PMC7705712; doi:10.1038/s41467-020-19891-7)
Supplement: Supplementary file 1 — Supplementary Information [file 41467_2020_19891_MOESM1_ESM.pdf]

SUPPLEMENTARY INFORMATION

Lethality of SARS-CoV-2 infection in K18 human angiotensin converting enzyme 2 transgenic mice

Authors: Fatai S. Oladunni[1] §, Jun-Gyu Park[1] §, Paula Pino Tamayo[1] §, Olga Gonzalez[1] §, Anwari Akhter[1] §, Anna Allué-Guardia[1] §, Angélica Olmo-Fontánez[1,2], Shalini Gautam[1], Andreu Garcia-Vilanova[1], Chengjin Ye[1], Kevin Chiem[1,2], Colwyn Headley[1], Varun Dwived[1]i, Laura M. Parodi[1], Kendra J. Alfson[1], Hilary M. Staples[1], Alyssa Schami[1,2], Juan I. Garcia[1], Alison Whigham[1], Roy Neal Platt II[1], Michal Gazi[1], Jesse Martinez[1], Colin Chuba[1], Stephanie Earley[1], Oscar H Rodriguez[1], Stephanie Davis Mdaki[1], Katrina N Kavelish[1], Renee Escalona[1], Cory R. A. Hallam[1], Corbett Christie[1], Jean L. Patterson[1], Tim J. C. Anderson[1], Ricardo Carrion Jr[1], Edward J. Dick Jr[1], Shannan Hall-Ursone[1], Larry S. Schlesinger[1], Xavier Alvarez[1], Deepak Kaushal[1], Luis D. Giavedoni[1], Joanne Turner[1,*], Luis Martinez-Sobrido[1,*] and Jordi B. Torrelles[1,*]

Affiliations: [1]Texas Biomedical Research Institute, San Antonio, TX, 78227, USA, and [2]Integrated Biomedical Sciences Program, University of Texas Health Science Center at San Antonio, TX, 78229, USA.

§ Authors contributed equally in this study

***Corresponding authors:** Dr. Luis Martinez-Sobrido, lmartinez@txbiomed.org; Dr. Jordi B. Torrelles, jtorrelles@txbiomed.org; and Dr. Joanne Turner, joanneturner@txbiomed.org.

Supplementary Table 1. Evaluation of SARS-CoV-2 infected K18 hACE2 transgenic C57BL/6 mice in Lung and Brain Severity Scored. When not indicated, observed in the majority of females and males

Mouse	DPI**	Lung	Brain		Mouse	DPI**	Lung	Brain		Mouse	DPI**	Lung	Brain
1	2	2	0		1	4	1	0		1	6	1	1
2	2	1	0		2	4	2	0		2	6	1	1
3	2	1	0		3	4	1	1		3	6	2	2
4	2	2	0		4	4	2	0		4	6	3	2
5	2	1	0		5	4	3	0		5	6	3	3
6	2	1	0		6	4	3	1		6	6	4	2

**Severity score for lung and brain overtime. 0 = normal; 1 = minimal (≤10%); 2 = mild (11-30%), 3 = moderate (30-60%), 4 = marked (60-80%), 5+ severe (>80%). DPI: Days post-infection. Different individual mice assessed (#1 to #6)

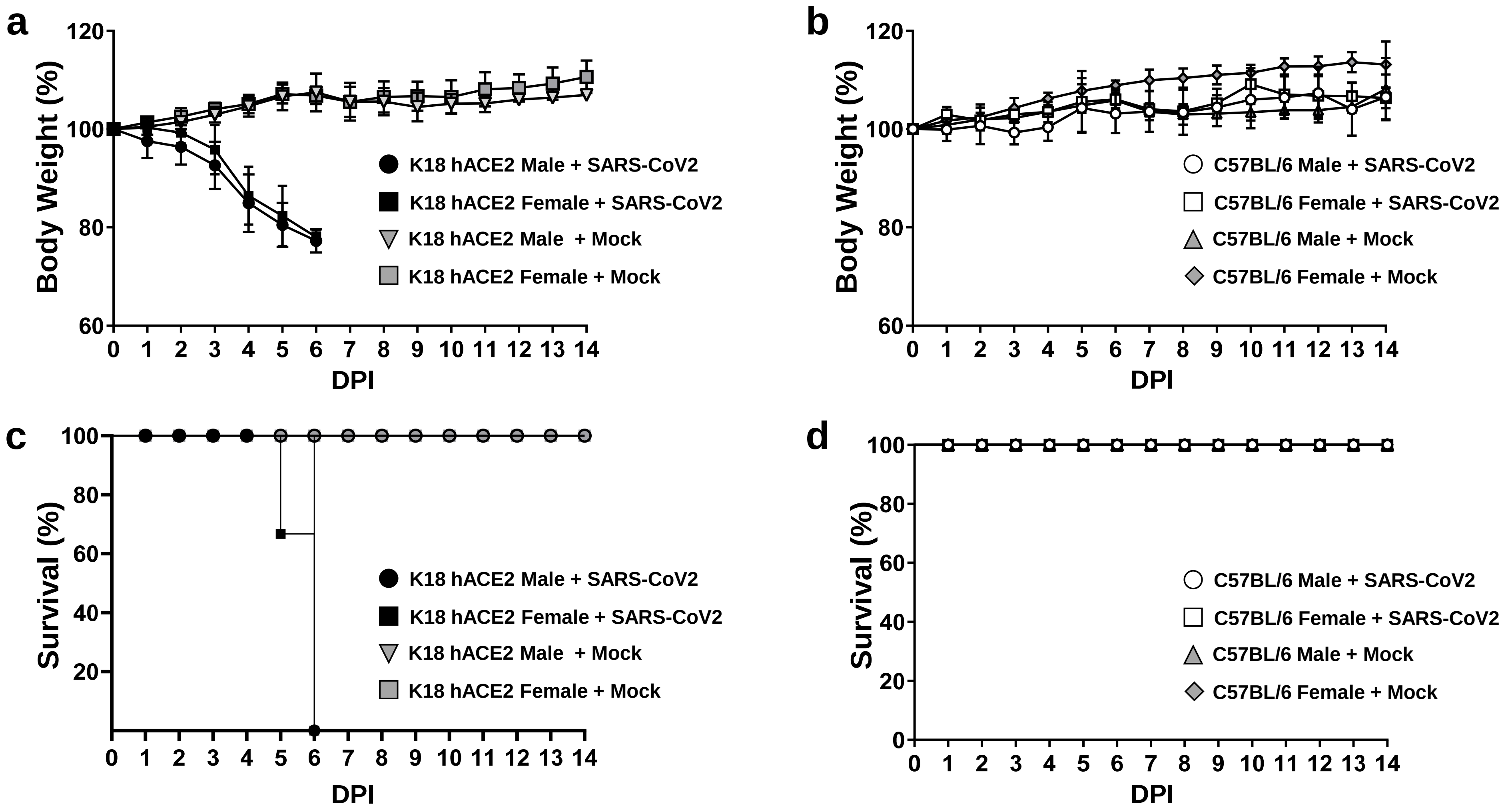

Supplementary Figure 1. Infection of K18 hACE2 transgenic and WT C57BL/6 mice with SARS-CoV-2. Male (n=7) and female (n=7) K18 hACE2 transgenic, and female (n=4) and male (n=4) WT C57BL/6 mice were infected or mock-infected (n=3/mouse strain/sex) i.n. with 1 x 10^5 PFU of SARS-CoV-2. Body weight (**a**, **b**) and survival (**c**, **d**) were evaluated at the indicated DPI. Mice that loss more than 25% of their initial body weight were humanely euthanized. Error bars represent standard deviations (SD) of the mean for each group of mice. DPI: Days post-infection. Data are combined from 2 independent experiments. Source data are provided as a Source Data file.

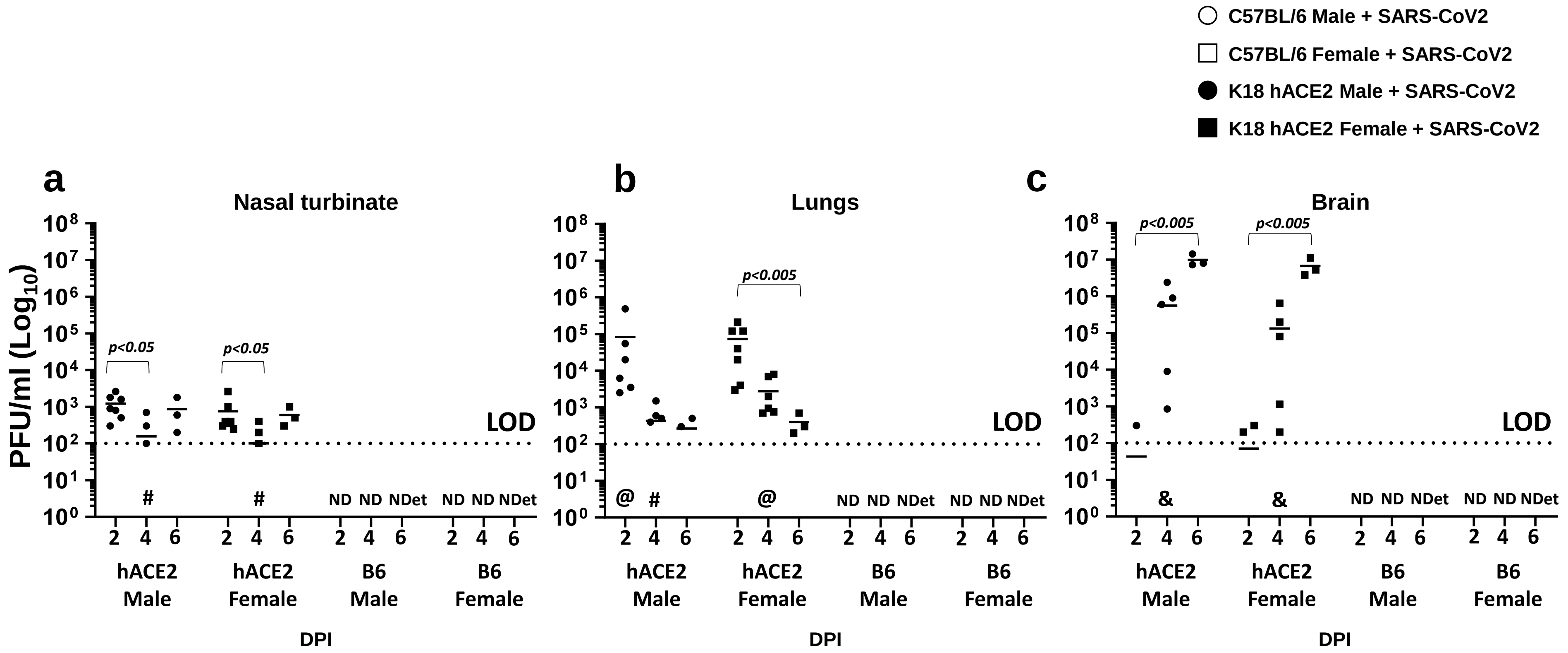

Supplementary Figure 2. Viral loads in male and female K18 hACE2 transgenic and WT C57BL/6 mice infected with SARS-CoV-2. K18 transgenic hACE2 male (n=17 mice distributed as n=7 for 2-DPI, n=7 for 4-DPI and n=3 for 6-DPI) and female (n=17 mice, distributed identically as the males) and WT C57BL/6 male (n=8 mice distributed as n=4 for 2-DPI and n=4 for 4-DPI) and female (n=8 mice distributed identically as the males) infected as in Figure S1 and sacrificed at 2- and 4-DPI and viral titers in different organs (nasal turbinate, trachea, lung, brain, heart, kidney, liver, spleen, small intestine, and large intestine) were determined by plaque assay (PFU/ml). Only data from virus containing organs and/or tissue samples are shown: nasal turbinate (**a**), lungs (**b**) and brain (**c**). Error bars represent standard deviations (SD) of the mean for each group of mice. Symbols represent data from individual mouse, and bars the geometric means of viral titers, $p < 0.05$; $p < 0.005$. @, virus not detected in one mouse; &, virus not detected in two mice; #, virus not detected in three mice; ND, not detected; NDet, non-determined. Dotted black lines indicate the limit of detection (10^2 PFU/ml). DPI: Days post-infection. Data are combined from of 2 independent experiments. Source data are provided as a Source Data file.

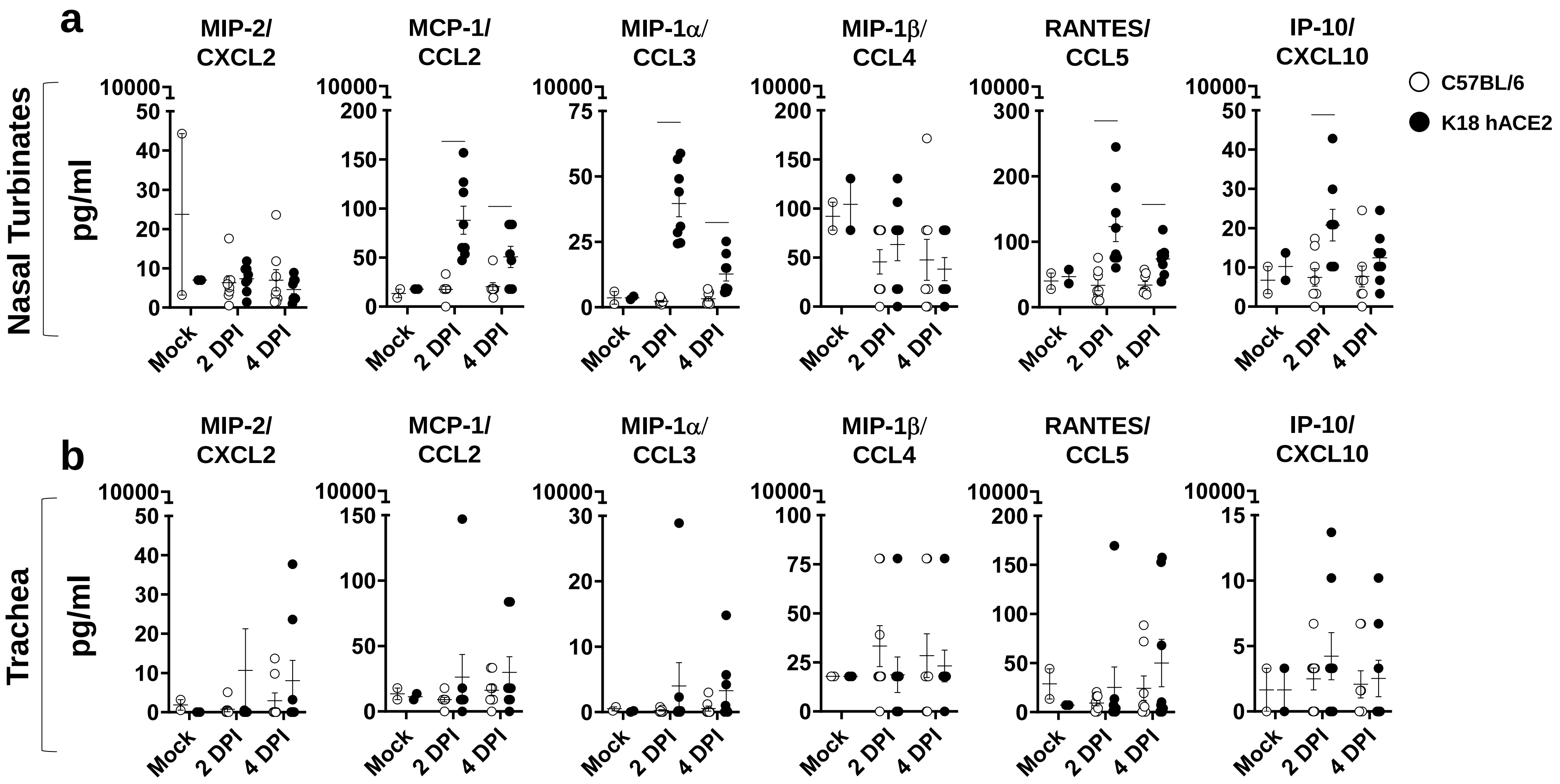

Supplementary Figure 3. Chemokine profile in selected tissues from SARS-CoV-2 infected K18 hACE2 transgenic mice. (**a**) Nasal turbinates and (**b**) Trachea. Student's *t*-test, two-tailed, C57BL/6 *vs.* K18 hACE2 $p< 0.05$; $p<0.005$; $p<0.0005$; 2-WAY ANOVA C57BL/6 or K18 hACE2 transgenic mice over time, $p< 0.05$; $p<0.005$; $p<0.0005$, M±SEM, n= 8 (per time-point studied, except mock n=2). DPI: Days post-infection. Data are representative over 2 independent experiments. Source data are provided as a Source Data file.

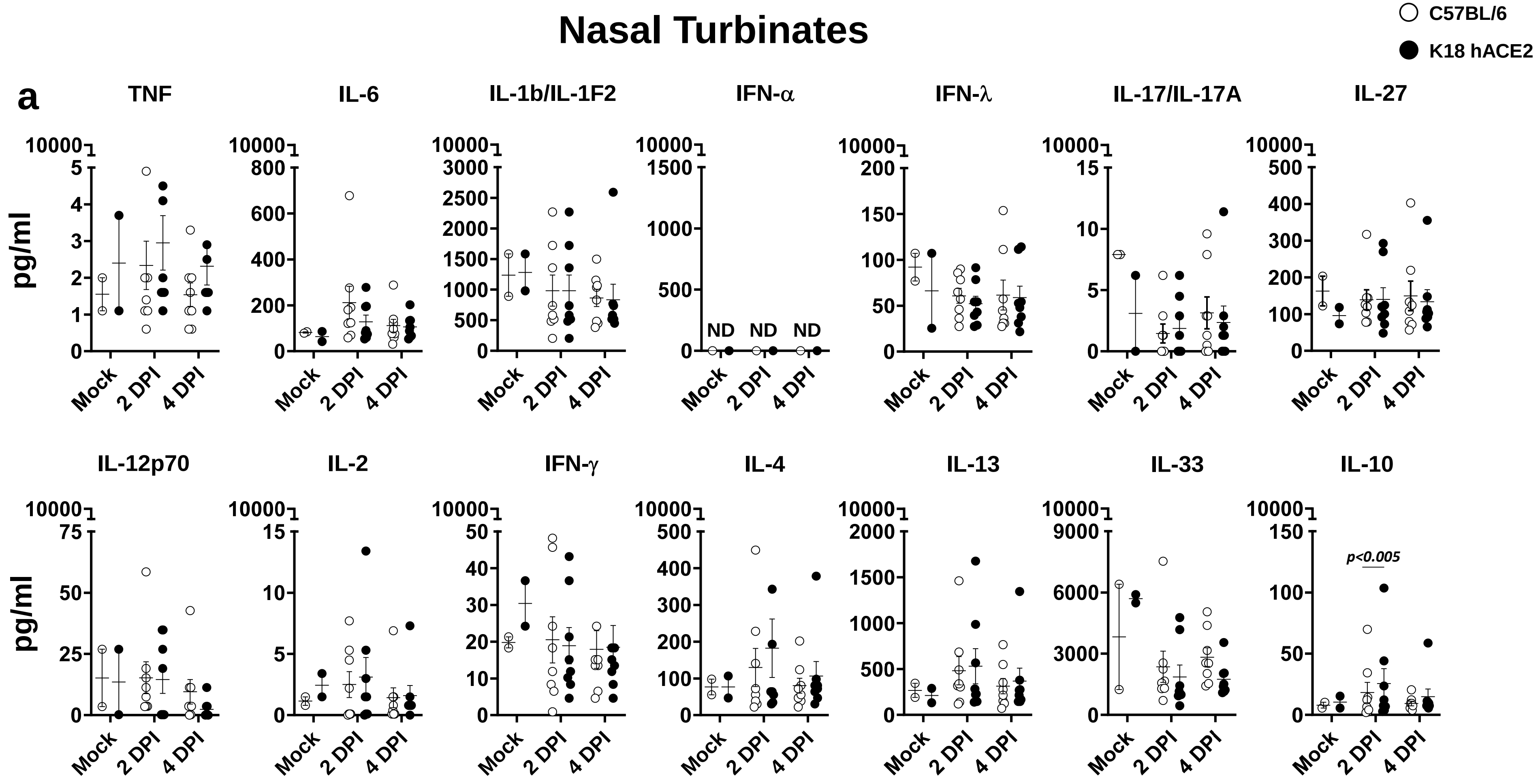

Supplementary Figure 4. Cytokine profile in selected tissues from SARS-CoV-2 infected K18 hACE2 transgenic mice. (**a**) Nasal turbinates and (**b**) Trachea. Student's t-test, two-tailed, C57BL/6 *vs.* K18 transgenic hACE2 $p < 0.05$; $p<0.005$; $p<0.0005$; 2-WAY ANOVA C57BL/6 or K18 hACE2 transgenic mice over time, $p < 0.05$; $p<0.005$; $p<0.0005$, M±SEM, n= 8 (per time-point studied, except mock n=2). DPI: Days post-infection. Data are representative over 2 independent experiments. Source data are provided as a Source Data file.

Trachea

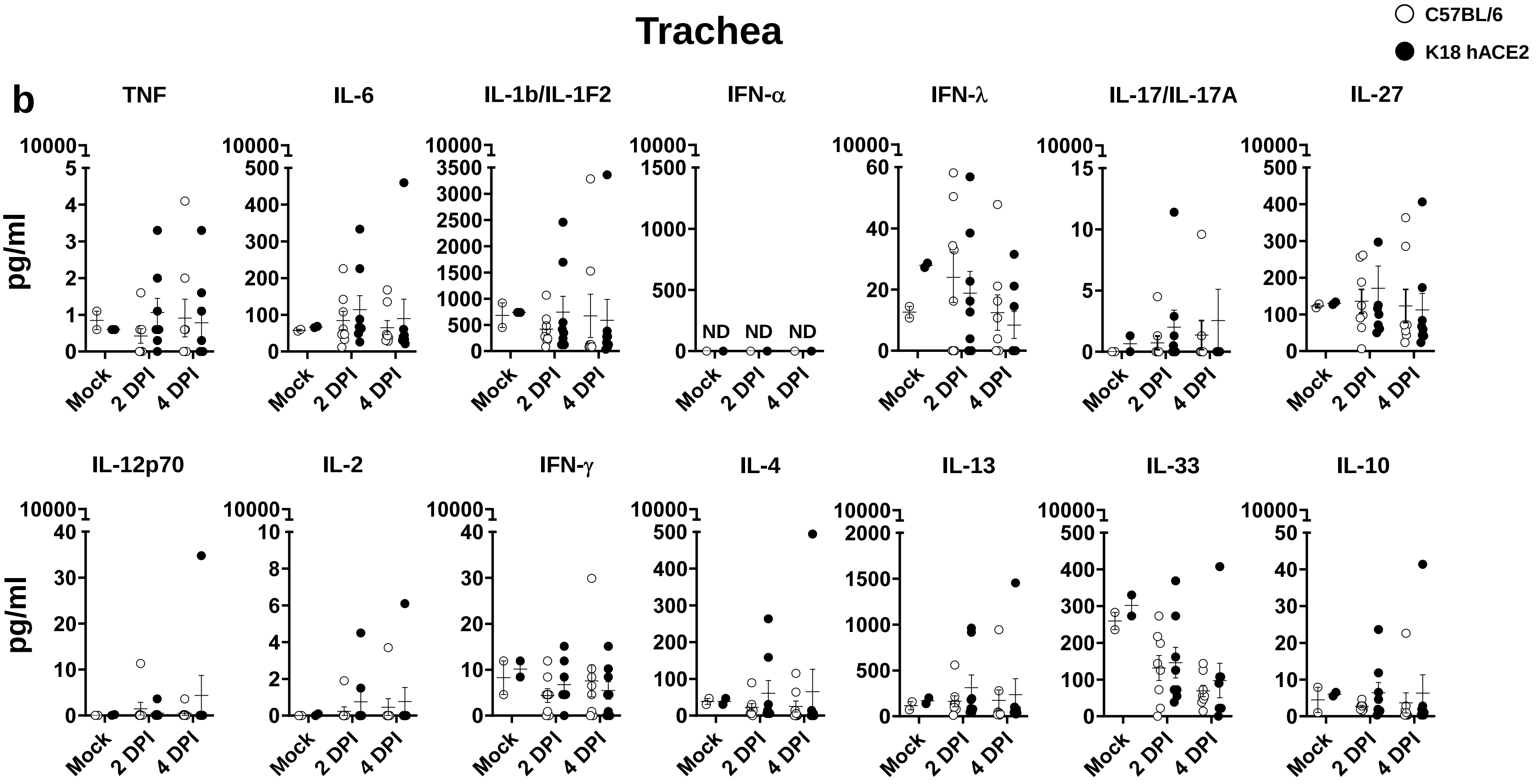

Supplementary Figure 4. Continuation.

Brain

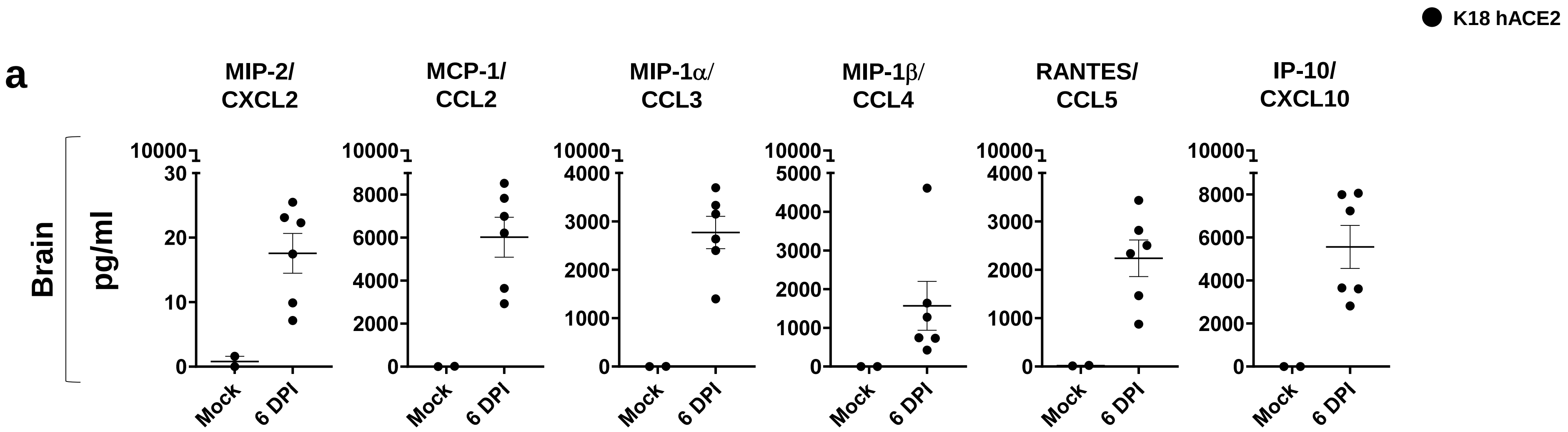

Supplementary Figure 5. Chemokines and cytokine profiles in brain tissues from SARS-CoV-2 infected K18 hACE2 transgenic mice at 6-DPI. (**a**) Chemokines and (**b**) Cytokines. M±SEM, n=6 (per time-point studied, except mock n=2). DPI: Days post-infection. Data are representative over 2 independent experiments. Source data are provided as a Source Data file.

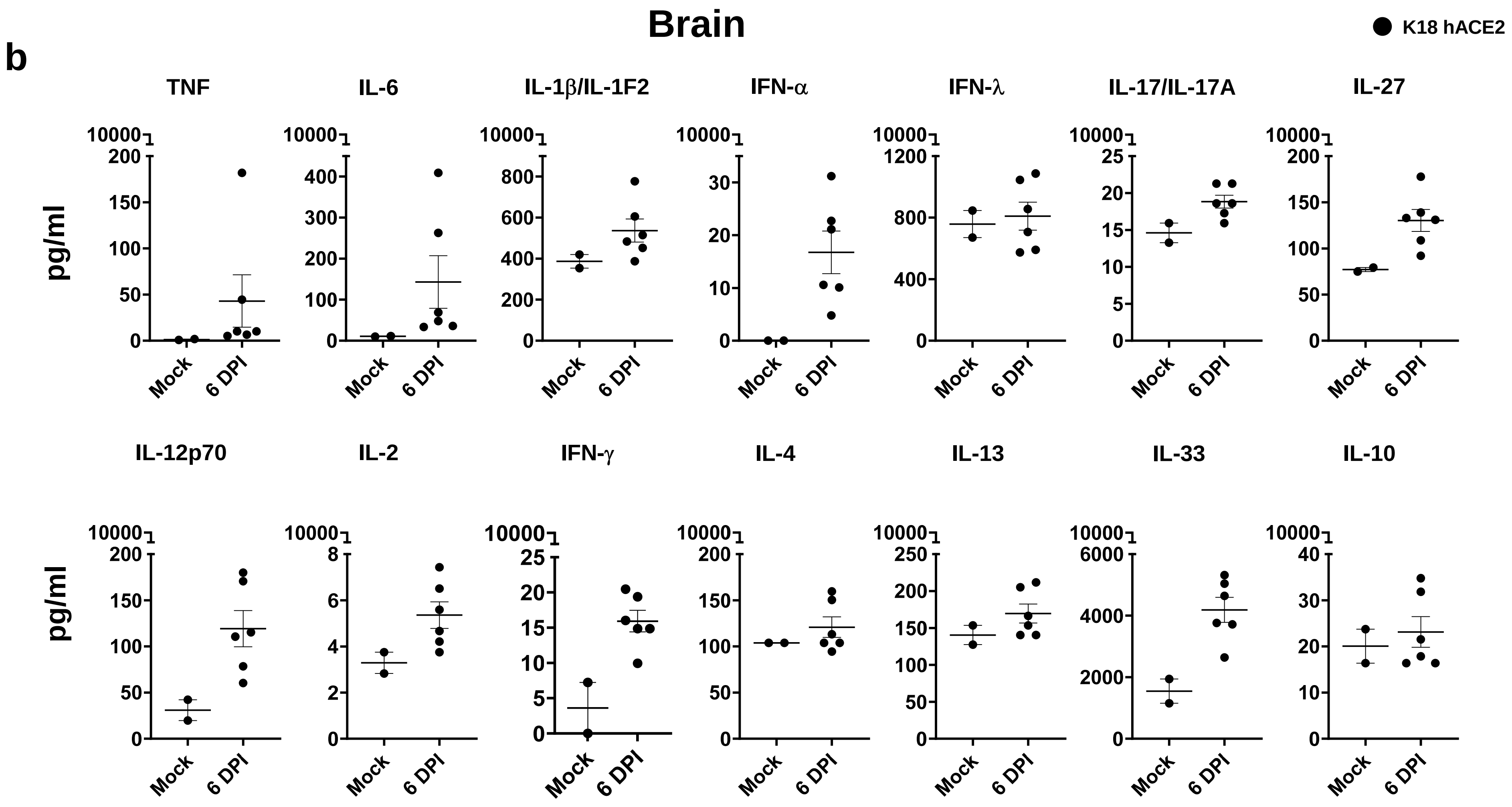

Supplementary Figure 5. Chemokines and cytokine profiles in brain tissues from SARS-CoV-2 infected K18 hACE2 transgenic mice at 6-DPI. (**a**) Chemokines and (**b**) Cytokines. M±SEM, n=6 (per time-point studied, except mock n=2). DPI: Days post-infection. Data are representative over 2 independent experiments. Source data are provided as a Source Data file.

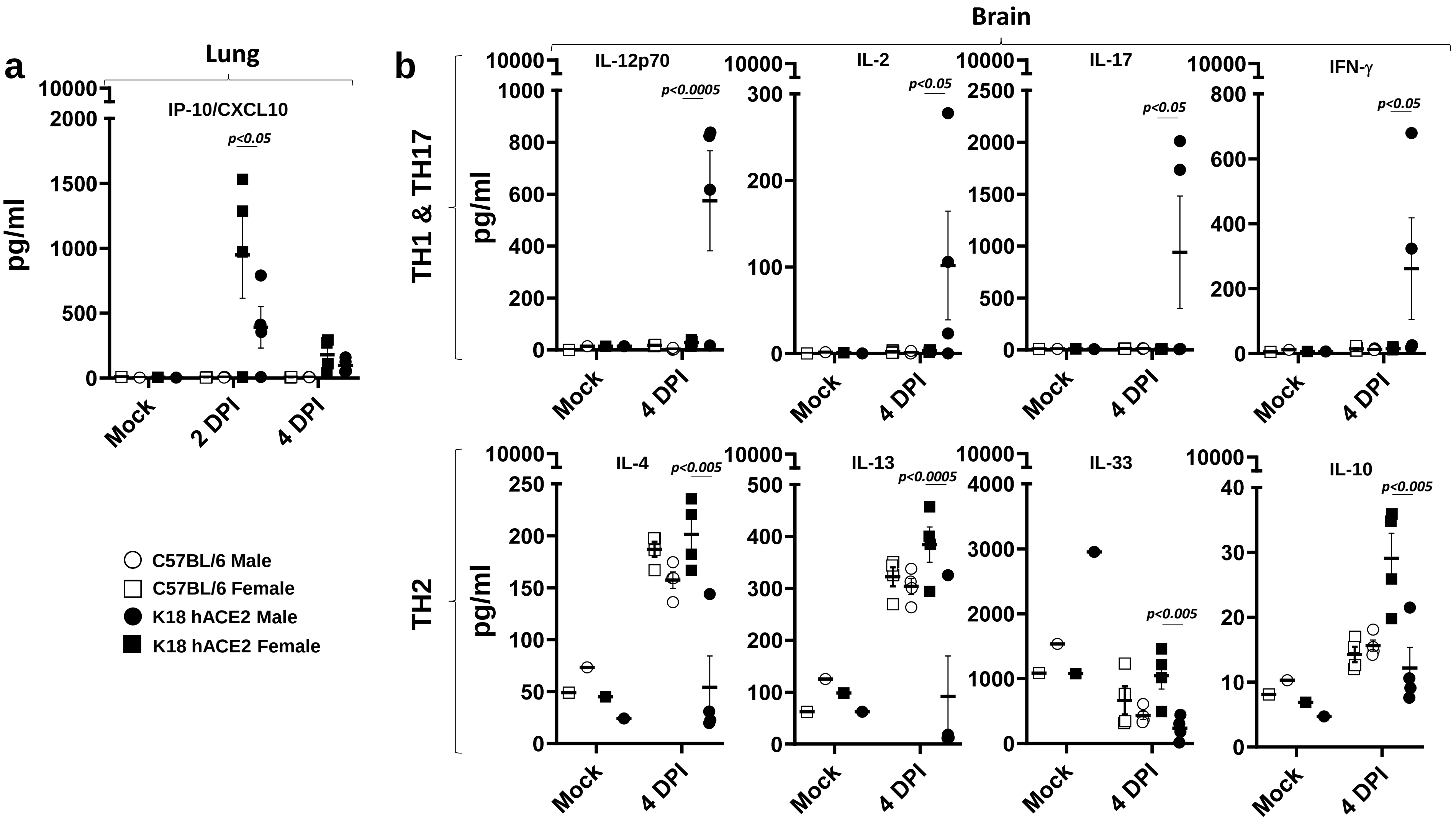

Supplementary Figure 6. Chemokine and cytokine profile in selected tissues from SARS-CoV-2 infected K18 hACE2 transgenic mice by sex. Chemokines and cytokine differences in the (**a**) lung and (**b**) brain in K18 hACE2 transgenic and WT C57BL/6 male and female mice. Student's t-test, two-tailed, C57BL/6 *vs.* K18 hACE2 $p < 0.05$; $p < 0.005$; $p < 0.0005$; 2-WAY ANOVA C57BL/6 or K18 hACE2 transgenic mice over time, $p < 0.05$; $p < 0.005$; $p < 0.0005$, M±SEM, n=4/group/sex (per time-point studied, except mock n=2). DPI: Days post-infection. Data are representative of 2 over independent experiments. Source data are provided as a Source Data file.

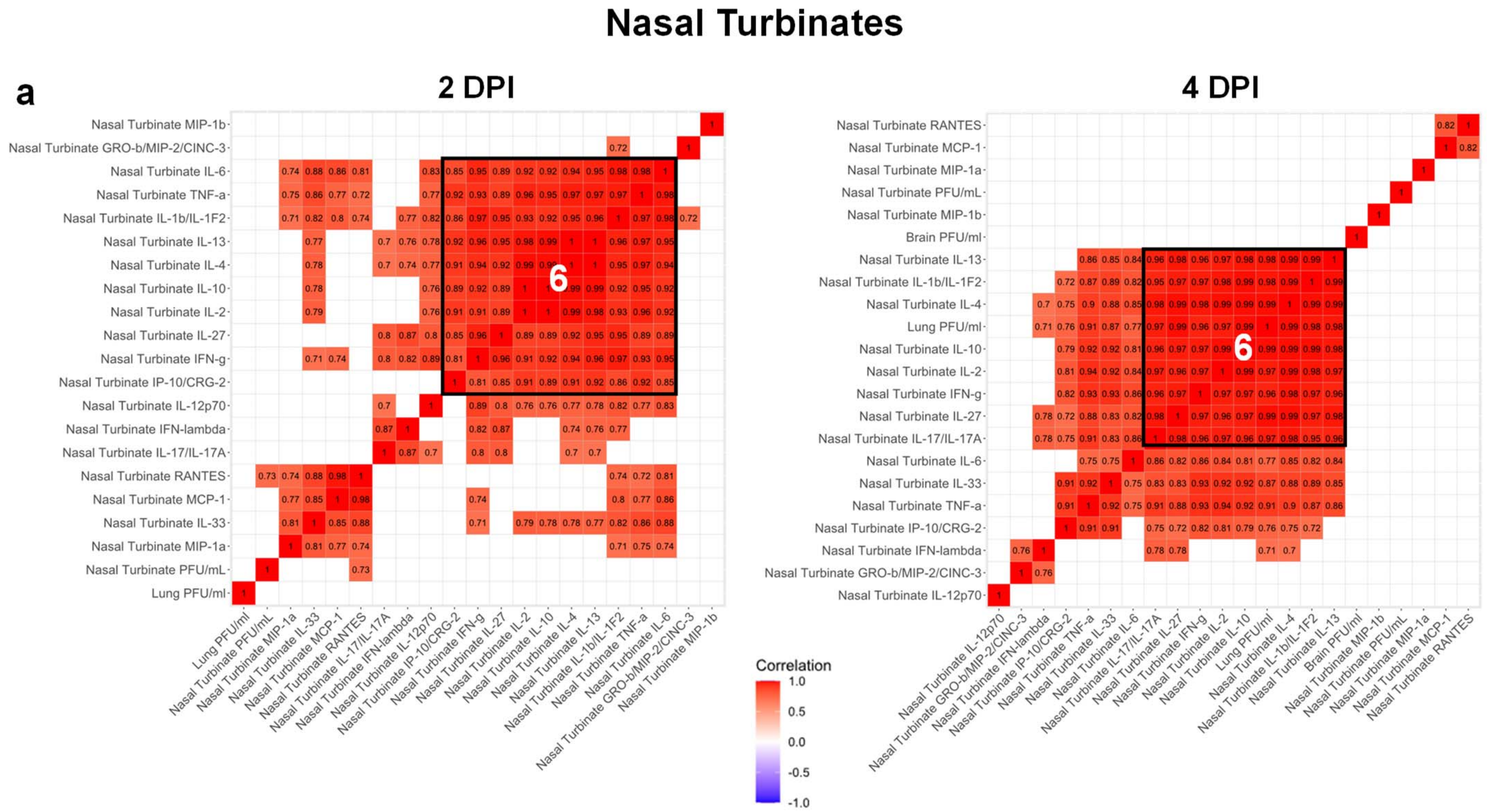

Supplementary Figure 7. SARS-CoV-2 infected K18 hACE2 transgenic mice reveal differentiated clusters of chemokine and cytokine correlations with clinical symptoms progression. Hierarchically clustered Pearson correlations of measurements in (**a**) nasal turbinate and (**b**) trachea of SARS-CoV-2 infected K18 hACE2 transgenic mice (Left: 2-DPI, Right: 4-DPI). Positive correlation (Red = 1) and negative correlations (Blue = -1), with clusters (Black outlined boxes with cluster number). Non-significant values ($p > 0.05$ measured by Pearson's correlation t-test) left blank. DPI: Days post-infection. MIP-2/CXCL2; MCP-1/CCL2; MIP-1α/CCL3; MIP-1β/CCL4; RANTES/CCL5; IP-10/CXCL10. Data are representative over 2 independent experiments. Source data are provided as a Source Data file.

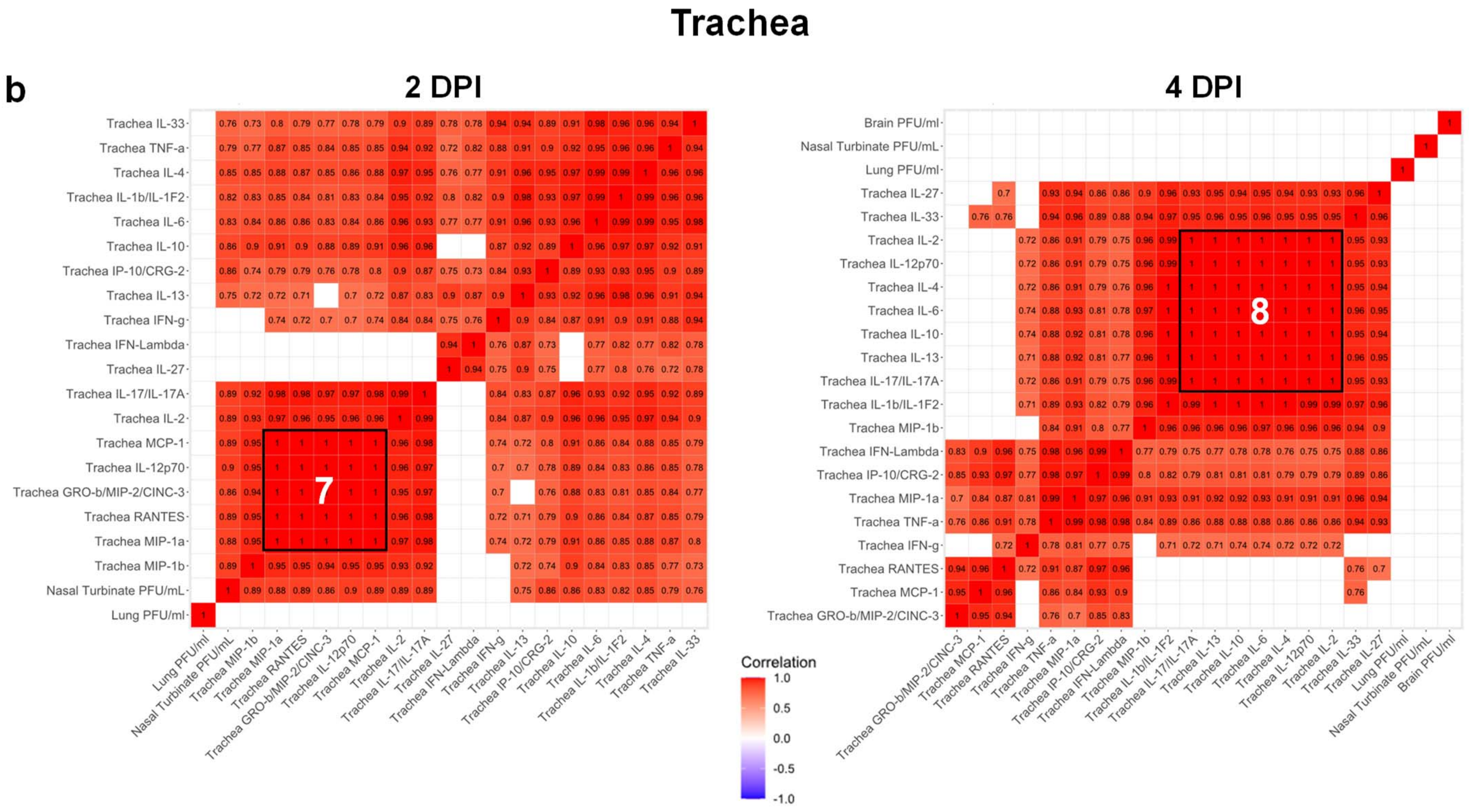

Supplementary Figure 7. Continuation.

Lungs

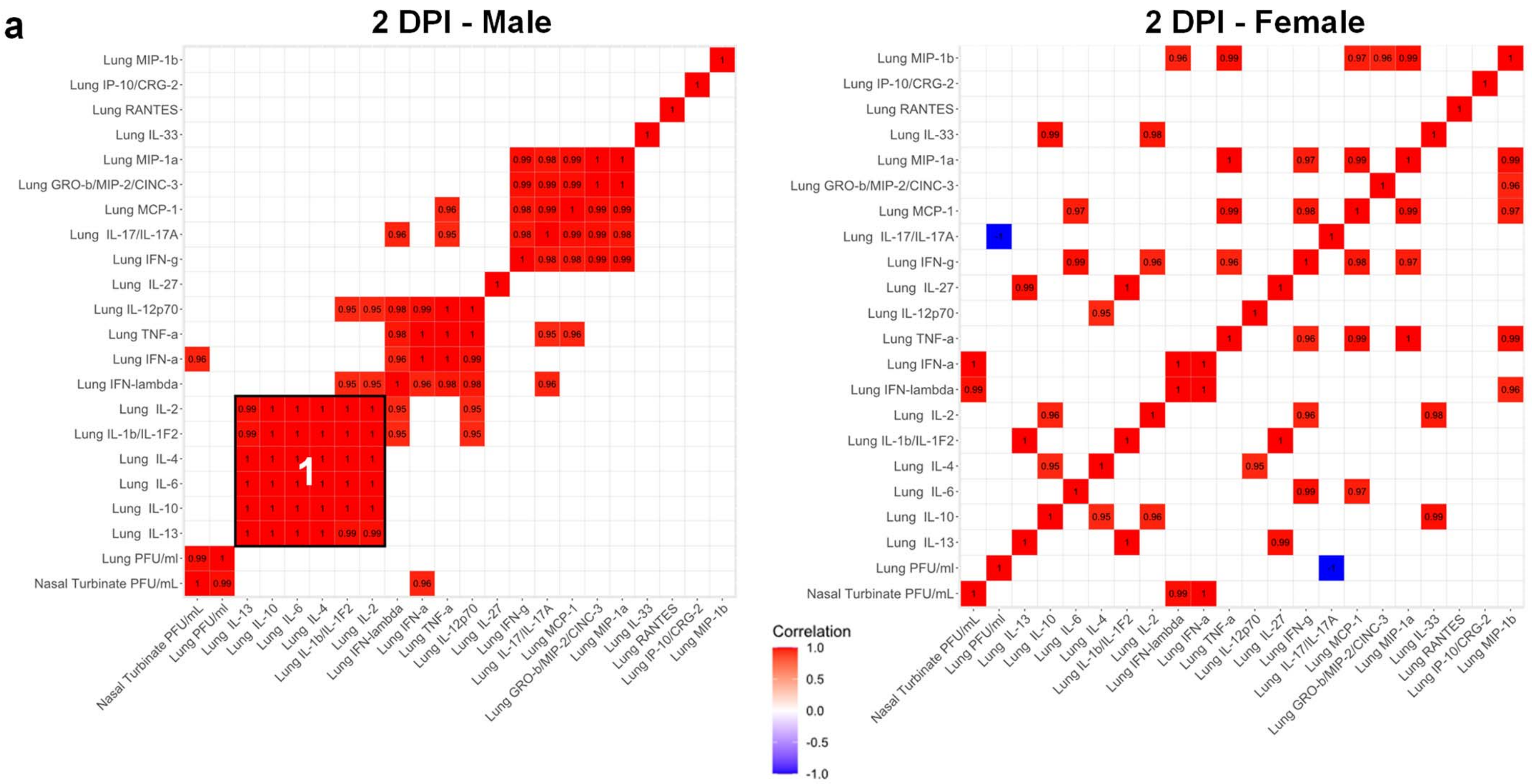

Supplementary Figure 8. **Immune response of SARS-CoV-2 infected K18 hACE2 transgenic mice is dependent on mouse sex.** Hierarchically clustered Pearson correlations of measurements in lung at (**a**) 2- and (**b**) 4-DPI and brain at (**c**) 2- and (**d**) 4-DPI of SARS-CoV-2 infected K18 hACE2 transgenic mice (Left: Males, Right: Females). Positive correlation (Red = 1) and negative correlations (Blue = -1), with clusters (Black outlined boxes with cluster number). Non-significant values (p > 0.05 measured by Pearson's correlation t-test) left blank. DPI: Days post-infection. MIP-2/CXCL2; MCP-1/CCL2; MIP-1α/CCL3; MIP-1β/CCL4; RANTES/CCL5; IP-10/CXCL10. Data are representative over 2 independent experiments. Source data are provided as a Source Data file.

Lungs

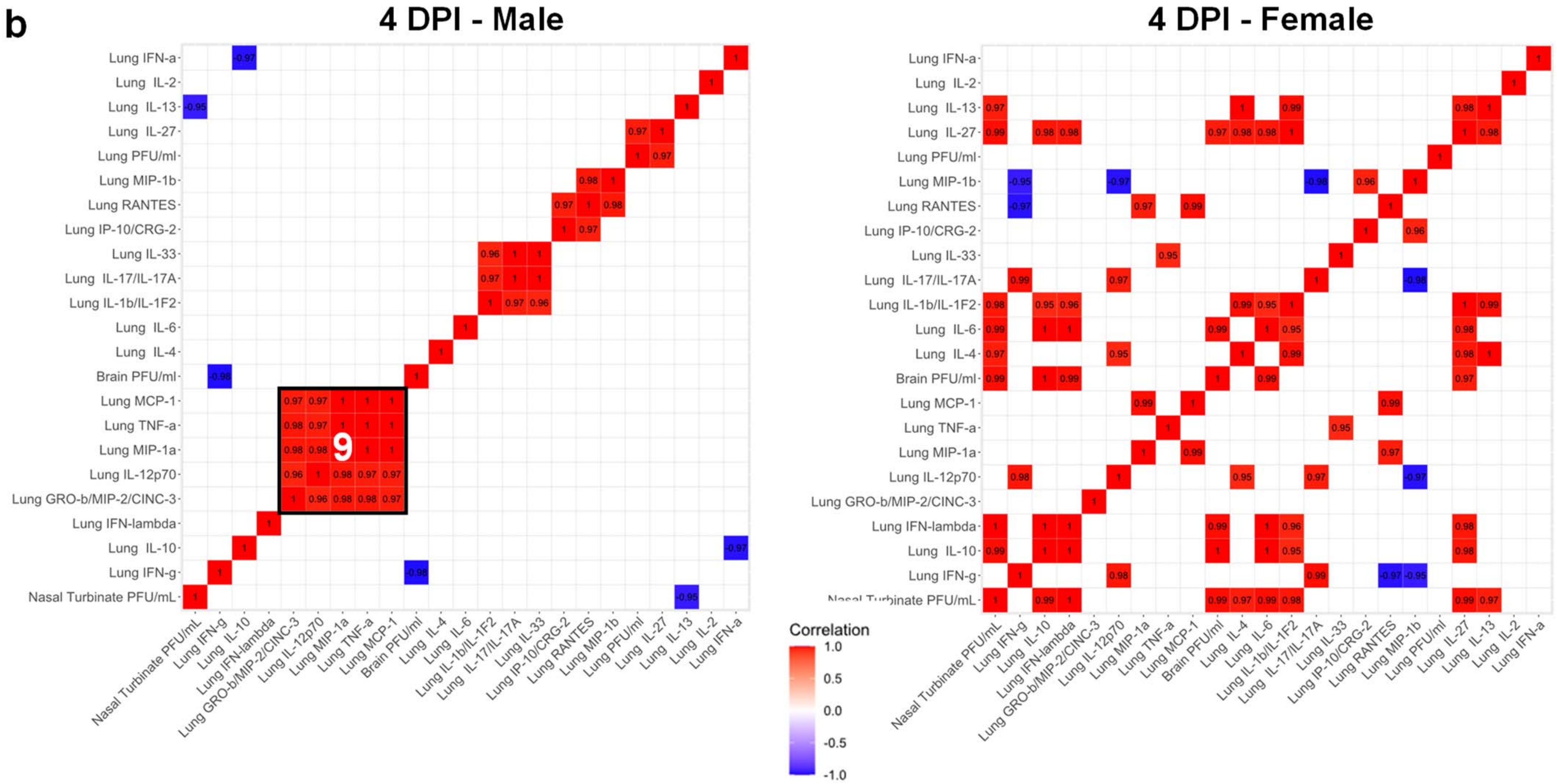

Supplementary Figure 8. Continuation.

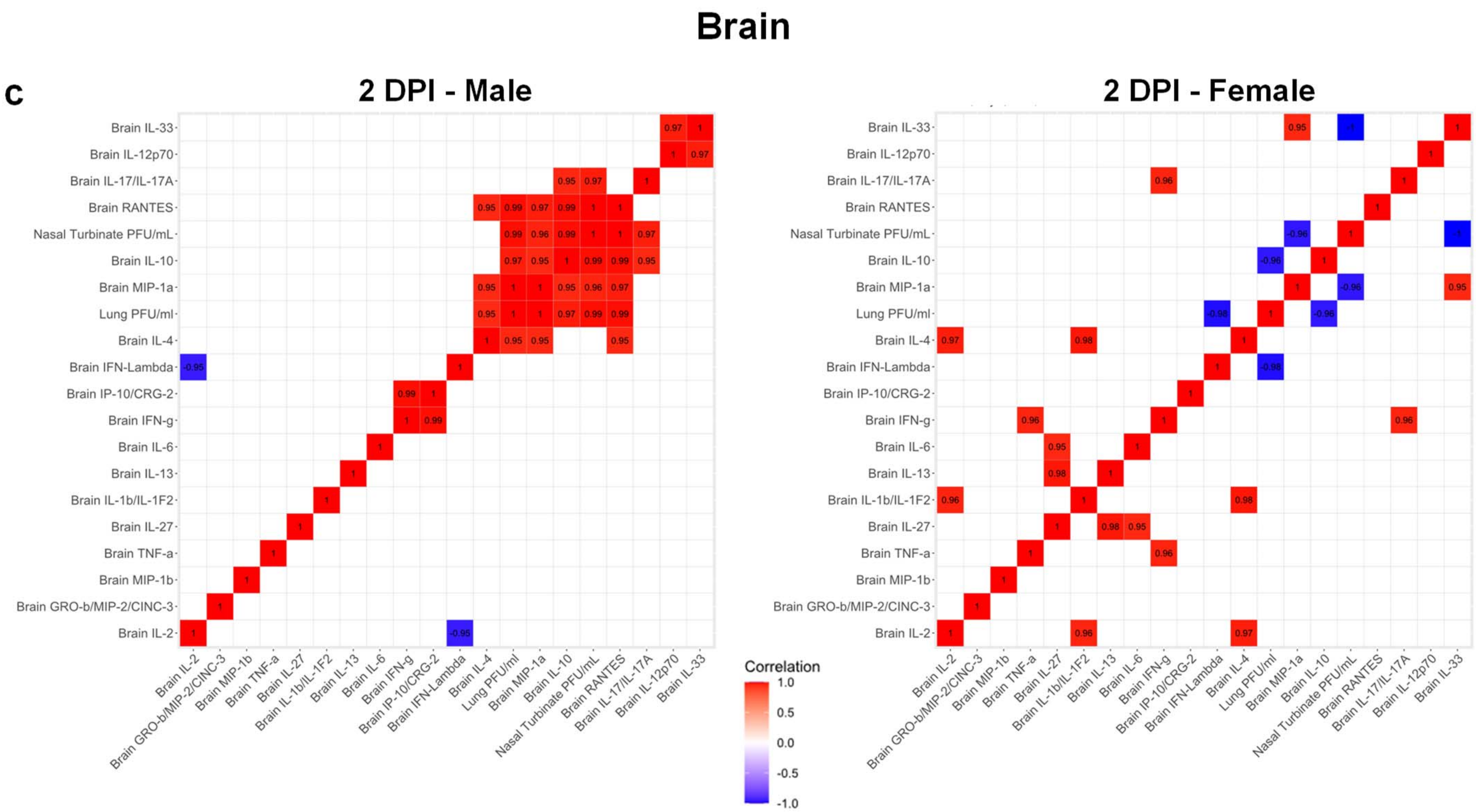

Supplementary Figure 8. Continuation.

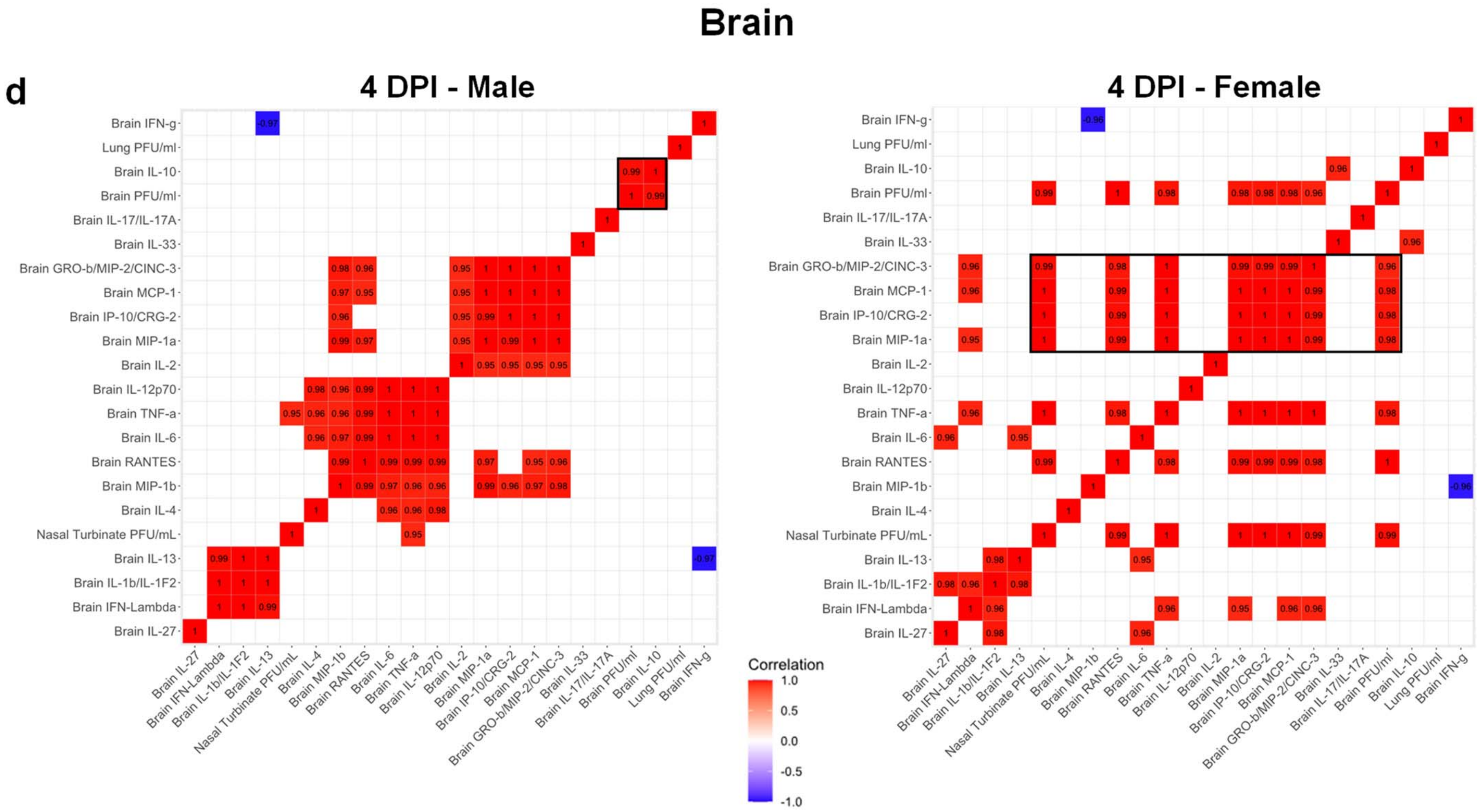

Supplementary Figure 8. Continuation.

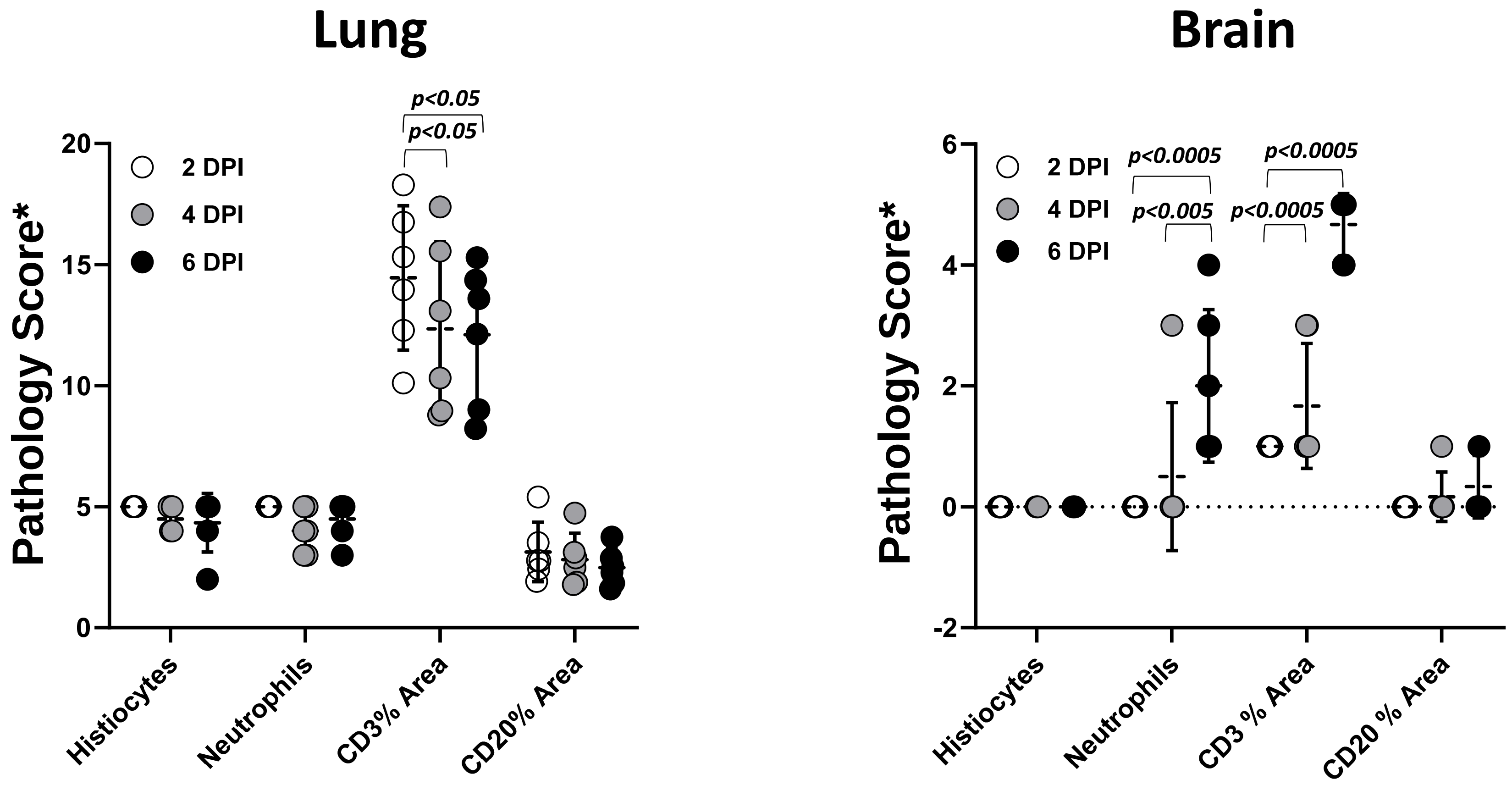

Supplementary Figure 9. Pathology score of immune cell infiltration into lung and brain of SARS-CoV-2 infected K18 hACE2 transgenic mice. Histiocytes and neutrophils were graded for severity according to the follow scores: 0=normal, 1=<10 cells, 2= <25 cells, 3= <50 cells, 4= >100 cells, 5=too many to count and plotted as a pathology score. $CD3^{+}$ and $CD20^{+}$ cells were graded by area of lung tissue involvement. 2-WAY ANOVA K18 hACE2 transgenic mice over time, $p < 0.05$; $p<0.005$; $p<0.0005$, M±SD, n = 6 (50:50 male:female per time-point and group) randomly chosen. Data are combined from of 2 independent experiments. Source data are provided as a Source Data file.
