# Supplementary material for: Lethality of SARS-CoV-2 infection in K18 human angiotensin-converting enzyme 2 transgenic mice
Source: Nat Commun. 2020 Nov 30;11:6122. doi: 10.1038/s41467-020-19891-7 (PMC7705712; doi:10.1038/s41467-020-19891-7)
Supplement: Supplementary file 3 — Reporting Summary [file 41467_2020_19891_MOESM3_ESM.pdf]

nature research

Corresponding author(s): Drs. Joanne Turner, Luis Martinez-Sobrido and Jordi B. Torrelles

Last updated by author(s): Nov 3, 2020

Reporting Summary

Nature Research wishes to improve the reproducibility of the work that we publish. This form provides structure for consistency and transparency in reporting. For further information on Nature Research policies, see our Editorial Policies and the Editorial Policy Checklist.

Statistics

For all statistical analyses, confirm that the following items are present in the figure legend, table legend, main text, or Methods section.

n/a	Confirmed	
☐	☒	The exact sample size (*n*) for each experimental group/condition, given as a discrete number and unit of measurement
☐	☒	A statement on whether measurements were taken from distinct samples or whether the same sample was measured repeatedly
☐	☒	The statistical test(s) used AND whether they are one- or two-sided<br>*Only common tests should be described solely by name; describe more complex techniques in the Methods section.*
☒	☐	A description of all covariates tested
☒	☐	A description of any assumptions or corrections, such as tests of normality and adjustment for multiple comparisons
☐	☒	A full description of the statistical parameters including central tendency (e.g. means) or other basic estimates (e.g. regression coefficient) AND variation (e.g. standard deviation) or associated estimates of uncertainty (e.g. confidence intervals)
☐	☒	For null hypothesis testing, the test statistic (e.g. *F*, *t*, *r*) with confidence intervals, effect sizes, degrees of freedom and *P* value noted<br>*Give P values as exact values whenever suitable.*
☒	☐	For Bayesian analysis, information on the choice of priors and Markov chain Monte Carlo settings
☒	☐	For hierarchical and complex designs, identification of the appropriate level for tests and full reporting of outcomes
☐	☒	Estimates of effect sizes (e.g. Cohen's *d*, Pearson's *r*), indicating how they were calculated

Our web collection on statistics for biologists contains articles on many of the points above.

Software and code

Policy information about availability of computer code

Data collection	Viral Determinations were acquired using C.T.L. Immunospot v7.0.15.0 Professional Analysis DC.<br>Cytokine and Chemokine data were acquired on a Luminex 100/200 System running on Xponent v4.2.<br>IFNs Type I and III were measure on a GloMax instrument running Glomax Explorer software v3.0.<br>Confocal microscopy images were acquired using Zeiss LSM 800 microscope running Zen Blue Edtion v2.1 software.<br>For Histopathology, images were acquired using an Olympus Bx46 microscope and Olympus CellSense v1.18 Life science Imaging software.
Data analysis	GraphPad Prism v8.0 was used for performing the data analysis of the results of this study and creating data plots.<br>Viral Determinations were analyzed using C.T.L. Immunospot v7.0.15.0 Professional Analysis DC.<br>For the correlation data, RStudio v1.2.5033 running R v4.0.2 and packages (ggplot2) v3.3.2 and (ggcorrplot) v0.1.3 was used.<br>For Luminex data, Millipore Sigma Belysa™ v1.0 was used.<br>For confocal microscopy data, Zeiss Zen Blue Edition v2.1 was used for any scale bars presented and image processing.<br>For visualization purposes, analyzed data was layed out in figures using Microsoft PowerPoint 2019 v1808 (build 10367.20048).

For manuscripts utilizing custom algorithms or software that are central to the research but not yet described in published literature, software must be made available to editors and reviewers. We strongly encourage code deposition in a community repository (e.g. GitHub). See the Nature Research guidelines for submitting code & software for further information.

Data

Policy information about availability of data

All manuscripts must include a data availability statement. This statement should provide the following information, where applicable:

- Accession codes, unique identifiers, or web links for publicly available datasets
- A list of figures that have associated raw data
- A description of any restrictions on data availability

The authors declare that all data supporting the findings of this study are available within the paper and its Supplementary Information files and from the corresponding authors upon reasonable request. Source data are provided with this paper. The virus used in this study was fully sequenced using next generation sequencing (NGS) with the GenBank accession deposit number MT576563. The Source Data file includes raw data for Figs 1 to 4 and supplemental Fig S1 to S9.

Field-specific reporting

Please select the one below that is the best fit for your research. If you are not sure, read the appropriate sections before making your selection.

☒ Life sciences ☐ Behavioural & social sciences ☐ Ecological, evolutionary & environmental sciences

For a reference copy of the document with all sections, see nature.com/documents/nr-reporting-summary-flat.pdf

Life sciences study design

All studies must disclose on these points even when the disclosure is negative.

Sample size	Based on the limited number of K18 hACE2 transgenic mice from The Jackson Laboratory, n=7 each for female and male mice (14 total) were used for morbidity and mortality studies, while n=7 each for female and male mice (14 total) were used for viral titers at 2- and 4-DPI, and n=3 each for female and male (6 total) at 6-DPI were used for viral titers, respectively. An n=3 each for female and male K18 hACE2 transgenic mice (6 total) were used as mock-infected controls in the morbidity and mortality studies, and 1 female and 1 male mock infected for the viral determination studies. For C57BL/6 WT (The Jackson Laboratory), n=4 each for female and male mice (8 total) infected and n=3 each for female and male mice (6 total) mock-infected were used for morbidity and mortality, while n=4 each for female and male mice (8 total) infected were used for viral titers at 2- and 4-DPI, and 1 female and 1 male mock infected for viral determination. For chemokine/cytokine, histopathology and immunohistochemistry analyses, 7 female and 7 male for 2- and 4-DPI and 3 female and 3 male for 6-DPI SARS-CoV-2 infected; and 1 female and 1 male mock infected K18 ACE2 mice were used. As controls for these analyses, 4 female and 4 male SARS-CoV-2 infected for 2- and for 4-DPI, and 1 male and 1 female for mock WT C57BL/6 mice were used.<br><br>Cytokines and chemokines were performed in duplicate. Histopathology was performed in all animals.<br><br>IHC was performed in 8 K18 hACE2 transgenic mice (50:50 male:female, per time-point) and 2 WT mice (50:50 male:female, per time-point) randomly chosen.<br><br>Table 1 was generated from C57BL/6 WT n=8 (4 males and 4 females) for each time point 2- and 4-DPI, and K18 hACE2 transgenic n=8 (4 males and 4 females) for each time point 2- and 4-DPI, for a total of 32 mice. Table 2 was generated from K18 hACE2 transgenic mice only at 6-DPI n=6 (3 males and 3 females). Table 3 was generated from 3 time points 2-, 4- and 6-DPI, n=6 for each group/time-point. All animals were randomly chosen.
Data exclusions	No data were excluded
Replication	We performed two infections. Mortality and morbidity are shown as data combined of two independent experiments. Other studies are shown as a representative data of two independent experiments. All replication studies showed similar results and biological outcomes.
Randomization	Mice were randomly assigned to study groups (for viral load determination or survival) by vivarium personnel without any knowledge of the study aims. This study did not involve human or a clinical trial that require randomization.
Blinding	Group allocation was assigned randomly by vivarium personnel with no knowledge of the study design. All investigators acquiring the data were blinded, except for the investigator who performed the infections and acquired the data for the viral titers. Data were analyzed by different investigators without previous knowledge of the group and sample allocation.

Reporting for specific materials, systems and methods

We require information from authors about some types of materials, experimental systems and methods used in many studies. Here, indicate whether each material, system or method listed is relevant to your study. If you are not sure if a list item applies to your research, read the appropriate section before selecting a response.

Materials & experimental systems

n/a	Involved in the study	
☐	☒	Antibodies
☐	☒	Eukaryotic cell lines
☒	☐	Palaeontology and archaeology
☐	☒	Animals and other organisms
☒	☐	Human research participants
☒	☐	Clinical data
☒	☐	Dual use research of concern

Methods

n/a	Involved in the study	
☒	☐	ChIP-seq
☒	☐	Flow cytometry
☒	☐	MRI-based neuroimaging

Antibodies

Antibodies used

SARS-CoV-1/2 NP cross-reactive monoclonal antibody (MAb), 1C7, Sigma, ZMS-1075.
SARS-CoV-1/2 NP, a rabbit polyclonal antibody shown to cross-react with SARS-CoV-2 NP, in house made.
hACE2 Receptor (hACE2 Recombinant Rabbit Monoclonal Antibody, Thermo-Fisher, Cat#SN0754)
Goat Anti-Rabbit AlexaFluor 488 (goat anti rabbit-Alexa 488, Thermo-Fisher, Cat#A-11008)
CD3 (Dako/Agilent, Cat#A0542)
CD20 (Abcam, Cat#ab64088)

Validation

Validation of these antibodies has been performed by the manufacture companies. SARS-COV-1/-2 NP polyclonal antibody was validated in house by using C57BL/6 WT mice, and K18 hACE2 transgenic mice infected and mock infected using the antibody and isotype control.
SARS-CoV-1/2 NP cross-reactive monoclonal antibody (MAb), 1C7, Sigma, ZMS-1075.https://www.sigmaaldrich.com/catalog/product/sigma/zms1075?lang=en&region=US.Validated by the manufacturer and use with appropriate controls to ensure proper performance was consistent.
hACE2 receptor (https://www.thermofisher.com/antibody/product/ACE2-Antibody-clone-SN0754-Recombinant-Monoclonal/MA5-32307). Validated by the manufacturer and use with appropriate controls to ensure proper performance was consistent.
Goat anti-rabbit-AP developed with Permanent Red warp (https://biocare.net/product/mach-3-rabbit-ap-polymer-detection/). Validated by the manufacturer and use with appropriate controls to ensure proper performance was consistent.
CD3 (https://www.labome.com/product/Dako/A0452.html). Validated by the manufacturer and use with appropriate controls to ensure proper performance was consistent.
CD20 (https://www.biocompare.com/9776-Antibodies/549779-CD20-antibody-SP32-Carboxyterminal-end/?pda=9776|549779_0_0||1|ab64088). Validated by the manufacturer and use with appropriate controls to ensure proper performance was consistent.

Eukaryotic cell lines

Policy information about cell lines

Cell line source(s)

Vero C1008 (E6 clone) cells were obtained from ATCC®, CRL-1586™

Authentication

We frequently evaluate the morphologic appearance, differentiation and growth rate of the cells used in this study. Only cell lines that display the morphologic features of Vero C1008 as described by the manufacturer were used in our study.

Mycoplasma contamination

We confirm that cell lines used through-out this study does not have any microbial or mycoplasma contamination.

Commonly misidentified lines (See ICLAC register)

No misidentified cell line was used in any part of this study.

Animals and other organisms

Policy information about studies involving animals; ARRIVE guidelines recommended for reporting animal research

Laboratory animals

Specific-pathogen free, 4-5-weeks-old, female and male B6.Cg-Tg(K18-ACE2)2Prlmn/J (Stock No: 034860, K18 hACE2) hemi-zygotes, or wild-type (WT) C57BL/6 control mice, were purchased from The Jackson Laboratory (Bar Harbor, ME). Mice were maintained under a 12 h light/dark cycle with lights on at 07:00 h and lights off at 19:00 h, in groups of 4 mice per cage for the end-points experiments, and 5 mice per cage for the morbidity and mortality studies in individually ventilated cages on a ventilated rack (Tecniplast, SA, Italy). The animal holding room is temperature and humidity controlled to 68-79°F and 30-70 % RH.

Wild animals

The study does not contain wild animals.

Field-collected samples

The study does not contain samples collected from the field.

Ethics oversight

All experimental procedures with animals were approved by the Texas Biomedical Research Institte (Texas Biomed) Insttutional Biosafety Committee (IBC, #20-004 and #20-010) and Institutional Animal Care and Use Committee (IACUC, #1708 MU) and under Biosafety Level 3 (BSL3) and Animal BSL3 (ABSL3) facilities at Texas Biomed.

Note that full information on the approval of the study protocol must also be provided in the manuscript.
